# Sarcomatoid Carcinoma of the Breast: An Unusual Clinical Presentation

**DOI:** 10.7759/cureus.52696

**Published:** 2024-01-21

**Authors:** Abhilasha Bhargava, Suraj Agrawal

**Affiliations:** 1 Department of Surgery, Jawaharlal Nehru Medical College, Datta Meghe Institute of Higher Education and Research, Wardha, IND

**Keywords:** breast pathology, spindle cell carcinoma, sarcomatoid, breast cancer, metastatic carcinoma

## Abstract

Breast sarcomatoid carcinoma, which are malignant tumors that form from the mesenchymal tissue of the mammary gland, are extremely uncommon and come in two varieties: primary and secondary (development associated to therapy). Breast sarcomas are malignancies that are aggressive and have a bad prognosis. Multidisciplinary surgery, chemotherapy, and radiotherapy are among the treatment possibilities. This case report reports a case of 35-year-old female presented to our hospital with a palpable tumor in her right breast and pungent discharge from her nipples. These signs persisted for over five years. A sarcomatoid breast cancer was discovered by postoperative histology.

## Introduction

Sarcomatoid carcinoma can be defined as an uncommon type of malignant tumor that is observed in different organs, such as the kidney, lung, esophagus, breast, and prostate [1]. Metaplastic carcinomas are reported in <1% of breast cancer cases as an uncommon form of the disease. Although it has a clinical and radiological look similar to other kinds of breast cancer, it is distinguished by rapid development and poor prognosis [2]. Immunohistochemistry (IHC) enables the diagnosis of metaplastic malignancy. The ideal approach for treating metaplastic cancer is not universally accepted, and most guidelines recommend treatment based on the lines of infiltrative ductal carcinoma [3]. Metaplastic carcinoma has a poorer overall survival rate than invasive ductal carcinoma. Patients whose breast mass is fast expanding should be examined for the possibility of metaplastic or sarcomatoid cancer.

## Case presentation

**Figure 1:**
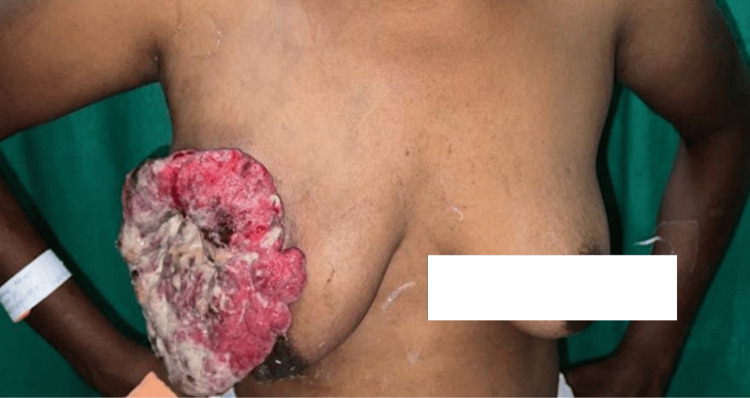
Clinical presentation of ulceroproliferative growth on the right breast

A 35-year-old woman complained of a lump in her right breast, present for five years. Initially insidious in onset with a 4 x 5 cm dimension, it rapidly started progressing in the last three months and reached its current dimensions of 10 x 15 cm with skin ulceration and discharge from the lump containing pus and blood (Figure 1). Post-op recovery was well, and no adverse events were noted. The patient was further subjected to palliative chemotherapy with adriamycin-cyclophosphamide regimen. Post chemotherapy, the patient was subjected to adjuvant radiotherapy.

**Figure 2:**
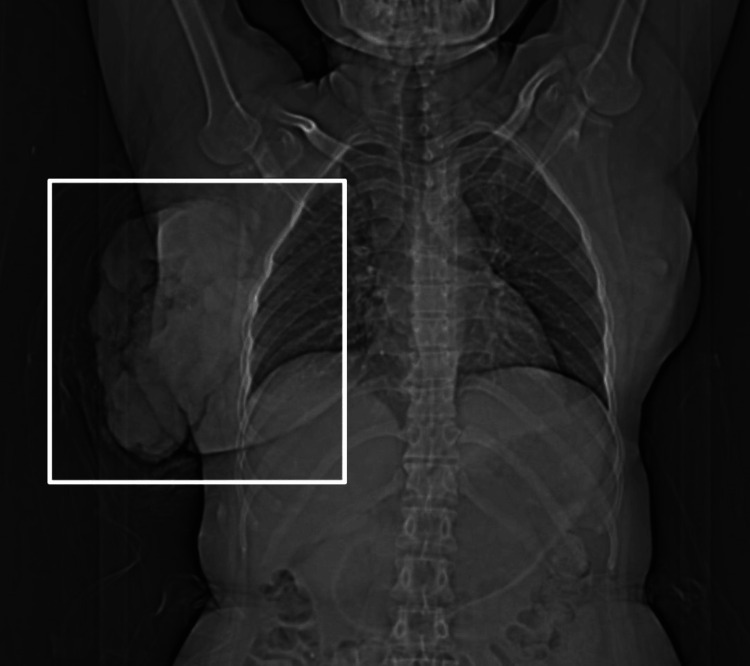
Chest radiograph depicting the growth over the right breast

It was distinctive by its foul odour discharge, everted edges, and cauliflower appearance. The patient had no significant family history of breast cancer or any other comorbidities. Sonography report of the breast with axilla was suggestive of a large heterogeneous echogenic mass with all quadrants involved in the right breast having increased vascularity and the presence of enlarged axillary lymph nodes, likely metastatic etiology (Figure 2).

**Figure 3:**
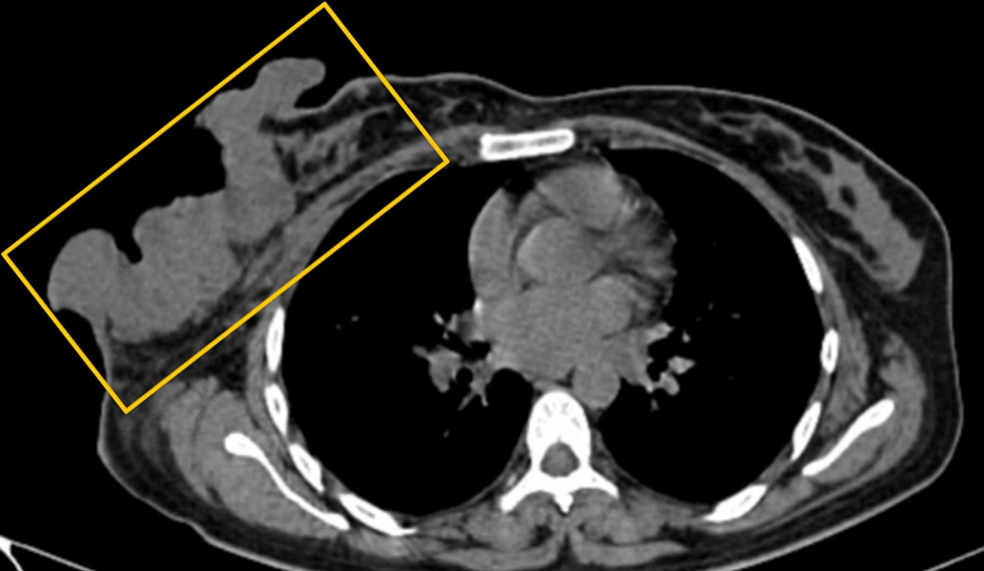
Computed tomography scan showing fungating growth invading the chest wall

The report of incisional biopsy of the lump over the right breast was suggestive of invasive ductal carcinoma. Ultrasound-guided fine needle aspiration cytology from the right axillary lymph node showed the presence of metastatic deposits. Metastatic workups for the chest, abdomen, and spine were performed by contrast-enhanced computerized tomography (CECT) thorax, which showed a large heterogeneously enhancing ill-defined irregular soft tissue density mass lesion in the retro-areolar region of the right breast involving the nipple-areolar complex. The lesion measured approximately 12.8 x 4.8 x 15.6 (AP x TR x CC) cm in size (Figure 3).

**Figure 4:**
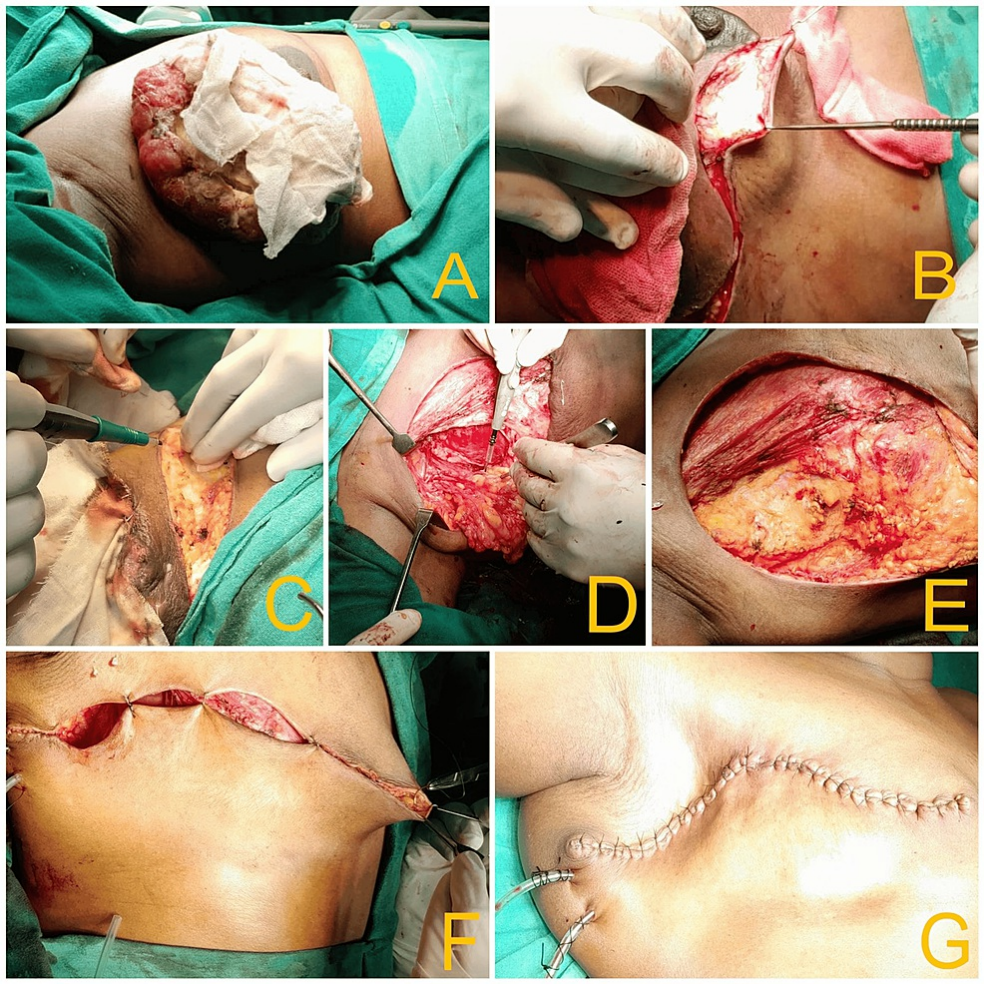
Intra-operative images of mastectomy A - after induction; B - starting with Stewart incision over the right breast; C - raising the flap; D - ipsilateral axillary clearance; E - post excision of the mass; F - initiating skin closure; G - post-op skin closure.

The lesion shows multiple non-enhancing necrotic areas with the underlying irregular hypodense non-enhancing collection and subcutaneous edema/fat stranding with prominent vasculature in the lesion. Anteriorly, the lesion involves subcutaneous tissue and skin with exophytic fungating growth; posteriorly, fat planes with pectoralis major muscle are lost at few planes, suggesting involvement. Subcutaneous edema/fat stranding was noted in the right posterior chest wall. Multiple sub-centimetric to centimetric lymph nodes were noted in the neck's right supraclavicular and posterior triangle. Multiple enlarged enhancing lymph nodes were also noted, a few of them adherent to each other in the right axilla, the largest with 1.2 x 1.7 x 1.9 cm dimensions. A few sub-centimetric lymph nodes were noted in the left axilla. The left breast appears normal. Palliative mastectomy, along with ipsilateral axillary dissection, was performed due to fungation and bleeding, and a section of the pectoralis major resected due to muscle infiltration by the tumor (Figure 4).

**Figure 5:**
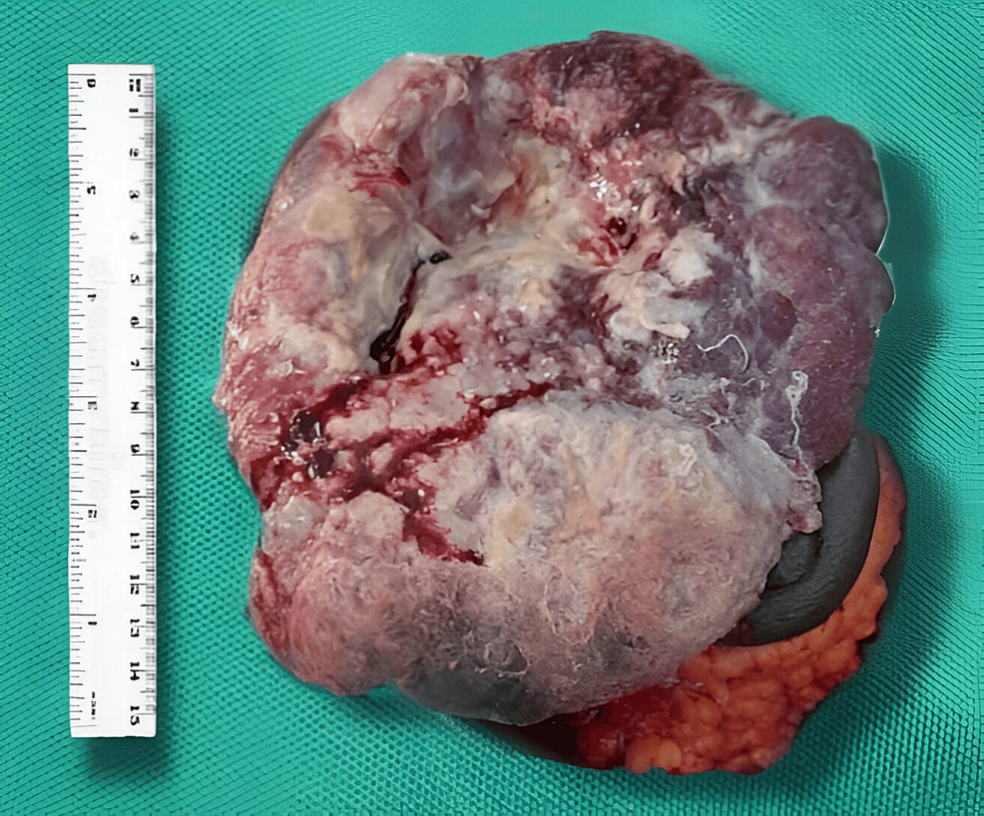
Excised specimen of the sarcomatoid carcinoma of the right breast

The excised specimen was sent for histopathology examination (Figure 5).

**Figure 6:**
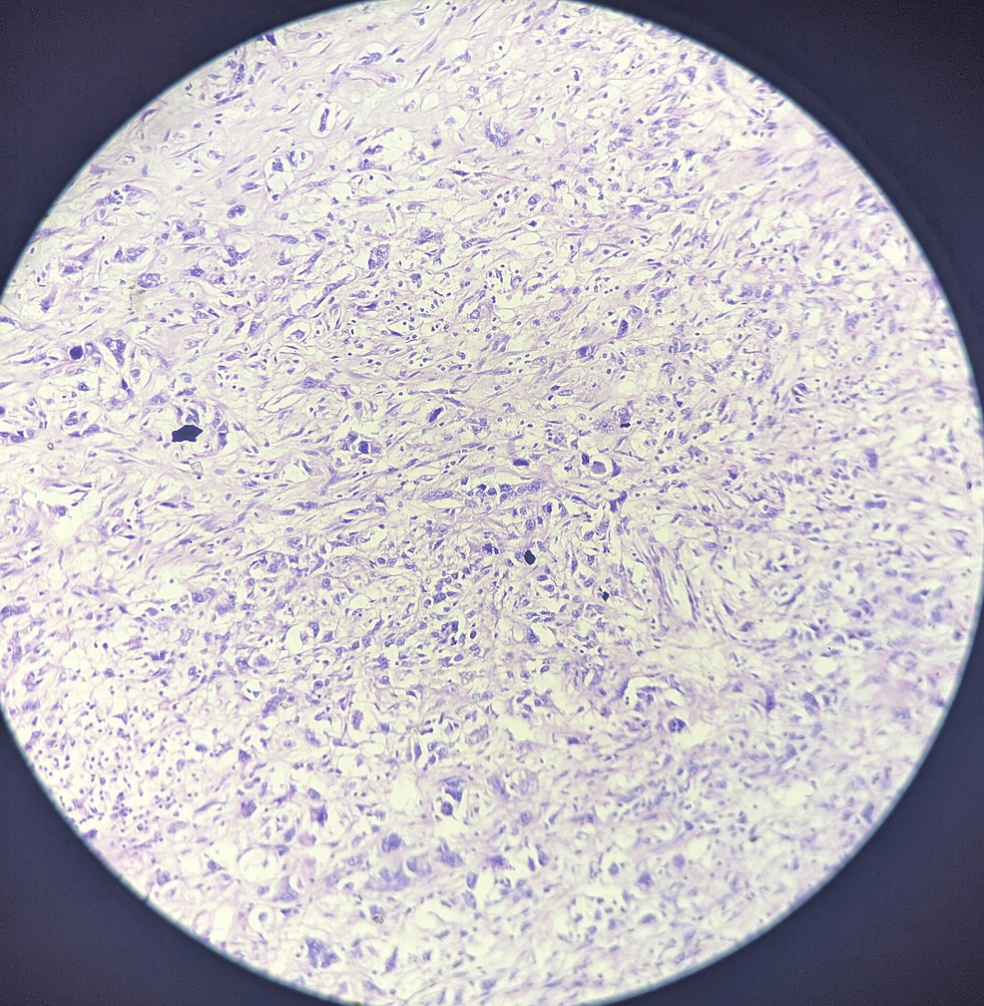
Histopathology slides showing the sarcomatoid breast carcinoma (10x magnification)

**Figure 7:**
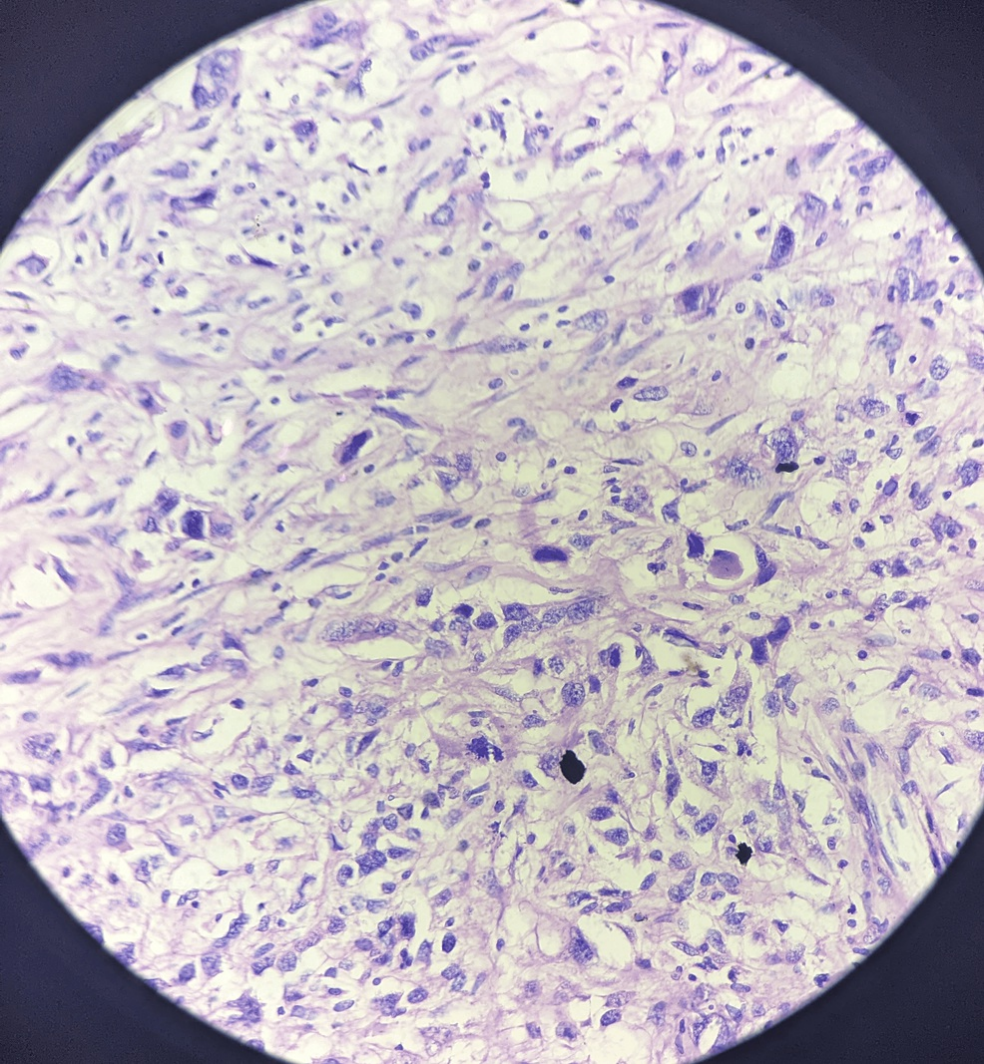
Histopathology slides showing the sarcomatoid breast carcinoma (40x magnification)

Immunohistochemistry of the patient revealed triple negative result with a zero score on ER scoring and PR scoring, and HER 2 was also reported negative. Ki67 was found to be high, i.e., 90%. During axillary dissection, 56 nodes were resected; all were found to be negative for metastasis. The histopathological features were suggestive of reactive lymphadenitis. The final histopathology report of the excised specimen of the breast was suggestive of metastatic carcinoma - sarcomatoid carcinoma (Figures 6, 7).

Post-op recovery was well, and no adverse events were noted. The patient was further subjected to palliative chemotherapy with adriamycin-cyclophosphamide regimen. Post chemotherapy, the patient was subjected to adjuvant radiotherapy. One month follow-up showed the patient recovering well. The scar site was healthy with no sign of infection and no fresh complaints, followed by palliative chemotherapy.

## Discussion

Breast metaplastic carcinomas are a diverse category of rare neoplasms that can be either monophasic (only sarcomatoid component) or biphasic (mixed carcinomatous and sarcomatoid components). Metastatic tumors have been divided into six types by the WHO Classification of Tumours of the Breast, 4th edition, namely, fibromatosis-like metaplastic carcinoma, low-grade adenosquamous carcinoma, spindle-cell carcinoma, squamous-cell carcinoma, metaplastic carcinoma with mesenchymal differentiation, myoepithelial carcinoma, and other types of or mixed mesenchymal differentiation [4]. Adenosquamous carcinomas and spindle cell carcinomas are examples of low-grade metaplastic carcinomas. Examples of high-grade metaplastic carcinomas are spindle-cell carcinoma and squamous cell carcinoma. Both clinically and radiographically, they present similarly to other breast cancers. Research reports have mentioned tumor presentation in 22 to 91 years [5-7]. At diagnosis, axillary lymph node metastasis was observed in 5-56% of cases. The lung and bone are the two most frequent locations for distant metastases. Original tumor differentiation and epithelium involvement can be considered the reason behind different levels of lymph node metastasis. Lymph node metastases are uncommon in primary breast sarcoma. Even when a pure spindle cell neoplasm is evident, the diagnosis of a malignancy must be considered if lymph nodes are positive. The incidence of axillary lymph node metastases in MBC cases is 6-26%. Research studies have mentioned that it is challenging to diagnose MBC accurately. Researchers have mentioned that only 11.8-20.0% of the patients had a correct diagnosis prior to mastectomy.

The mainstay of MBC treatment is surgery. Radiation and chemotherapy recommendations have been based on the disease stage and correlated factors. Both mortality rate and recurrence rate have been observed to be reduced with adjuvant radiation. The five-year overall survival rate reported in research for MBC was found to be 67.9% and 88.9%, respectively, compared to the IDC-not-defined type. The absence of intervening stroma and the presence of benign heterologous elements and carcinomatous elements can be utilized for a better prognosis. By contrast, tumor dimensions and sarcomatous metaplastic components, such as spindle cells and osteoid, cannot be efficiently used for a good prognosis [8]. Immunohistochemistry plays an essential part in determining a precise diagnosis of metaplastic carcinoma. It shows positive results for vimentin, smooth muscle actin, and pan-keratin; negative results are observed for progesterone receptor, estrogen receptor, and human epidermal growth factor receptor 2/ Neu protein in similarity in this case [5]. Because of its rarity and variety, the best treatment method for metaplastic carcinoma is unknown. Usually, IDC presents with sarcomatoid variation, but in this case, it was observed to be of mixed type. It is often treated similarly to a breast adenocarcinoma in most cases. In the majority of instances, the treatment consists of a mastectomy or broad local excision together with adjuvant chemotherapy [6]. Retrospective research has recommended that radiotherapy be included in the multimodal management of metaplastic carcinoma patients post surgery. In metaplastic carcinoma, epidermal growth factor receptors were more frequently expressed than conventional breast carcinoma. Based on these findings, they hypothesized that gefitinib or other protein kinase inhibitors would be more helpful in managing this aggressive tumor.

## Conclusions

Clinical management of a fast-growing breast tumor would require a high level of suspicion due to its rarity and also due to associated poor prognostic factors. Metaplastic carcinoma should be treated similarly to other invasive carcinomas because there is no specific therapy recommendation at this time. Due to the current research and the benefit of decreased sensitivity over traditional chemotherapy, patients can be greatly benefited in the coming future.
